# Nanotoxicologic Effects of PLGA Nanoparticles Formulated with a Cell-Penetrating Peptide: Searching for a Safe pDNA Delivery System for the Lungs

**DOI:** 10.3390/pharmaceutics11010012

**Published:** 2019-01-03

**Authors:** Larissa Gomes dos Reis, Wing-Hin Lee, Maree Svolos, Lyn Margaret Moir, Rima Jaber, Norbert Windhab, Paul Michael Young, Daniela Traini

**Affiliations:** 1 Respiratory Technology, Woolcock Institute of Medical Research and Discipline of Pharmacology, Faculty of Medicine and Health, The University of Sydney, Sydney, NSW 2037, Australia; lgom2879@uni.sydney.edu.au (L.G.d.R.); maree.svolos@sydney.edu.au (M.S.); lyn.moir@sydney.edu.au (L.M.M.); paul.young@sydney.edu.au (P.M.Y.); 2 Faculty of Pharmacy and Health Sciences, Universiti Kuala Lumpur-Royal College of Medicine Perak, (UniKL-RCMP), 30450 Ipoh, Perak, Malaysia; whlee@unikl.edu.my; 3 Evonik Industries AG, Kirschenallee, 64293 Darmstadt, Germany; Rima.jaber@evonik.com (R.J.); norbert.windhab@evonik.com (N.W.)

**Keywords:** alveolar, bronchial, apoptosis, autophagy, gene delivery

## Abstract

The use of cell-penetrating peptides (CPPs) in combination with nanoparticles (NPs) shows great potential for intracellular delivery of DNA. Currently, its application is limited due to the potential toxicity and unknown long-term side effects. In this study NPs prepared using a biodegradable polymer, poly(lactic–*co*–glycolic acid (PLGA) in association with a CPP, was assessed on two lung epithelial cell lines (adenocarcinomic human alveolar basal epithelial cells (A549) and normal bronchial epithelial cells (Beas-2B cells)). Addition of CPP was essential for intracellular internalization. No effects were observed on the mitochondrial activity and membrane integrity. Cells exposed to the NPs–DNA–CPP showed low inflammatory response, low levels of apoptosis and no activation of caspase-3. Increase in necrotic cells (between 10%–15%) after 24 h of incubation and increase in autophagy, induced by NPs–DNA–CPP, are likely to be related to the lysosomal escape mechanism. Although oxidative stress is one of the main toxic mechanisms of NPs, NPs–DNA–CPP showed decreased reactive oxygen species (ROS) production on Beas-2B cells, with potential antioxidant effect of CPP and no effect on A549 cells. This NP system appears to be safe for intracellular delivery of plasmid DNA to the lung epithelial cells. Further investigations should be conducted in other lung-related systems to better understand its potential effects on the lungs.

## 1. Introduction

Gene therapy has been rapidly advancing in recent years, and the commercial use of nanotechnology is increasing. Among the several uses of nanotechnology, nanomedicine has the potential of treating several diseases including cystic fibrosis, cancer, and other gene-related diseases [1]. The decrease in size into the nano-scale range provides novel physical and chemical properties to well-known materials. Although these unique properties offer a great potential for entering and transporting different materials into cells, its application has been limited due to the potential toxicity and unknown long-term side effects [2]. Careful measures need to be taken to ensure that no cellular dysfunction or harm has taken place following the administration of these nanoparticles (NPs).

Polymeric NPs show great potential in delivering nucleic acids to cells, due to their ability in encapsulating, increase in residence time, decrease in triggering immune response, and protecting pDNA from degradation. Cell-penetrating peptides (CPPs), which are able to enhance the internalization of these NPs in different cell lines [3], have also been used for this purpose. The use of poly(lactic–*co*–glycolic) acid (PLGA), a copolymer approved by the Food and Drug Administration (FDA) as therapeutic excipient, has been widely investigated for medical applications, mostly due to is biodegradability [4]. Although nanopolymeric systems have previously demonstrated promising results in the delivery of nucleic acid, this area has not been explored in depth, especially regarding the toxic effect of these formulations on the cells.

In general, the size, surface charge and general physicochemical properties of the NPs play a significant role in determining the toxic effects of these systems, which directly impacts on particles reactivity [5,6]. For instance, different coatings have been studied to decrease the reactivity of these particles. Furthermore, toxic effects have been associated with the induction of reactive oxygen species (ROS) production, as well as inflammation and damage to proteins, DNA and cellular membrane [6]. Disruption of cell cycle regulation has also been associated to the stress caused by NPs [7]. The differences in toxicity observed between macroparticles and NPs produced from the same materials [8] highlight the need to further investigate the effects of these NPs in vitro, prior to any further investigation in vivo.

Gene therapies include the use of a small circular DNA called plasmid DNA (pDNA), which offers, as the main advantages, its ease in isolation and manipulation, and its varied size and dynamic structure [9]. To achieve a successful gene delivery, pDNA needs to be internalized by the cell and proceed into the nucleus to get expressed. As most of the NPs get internalized by endocytosis, to avoid degradation by the endosomal–lysosomal system, the NPs have to escape from the vesicles and be released into the cytosol [10]. During the process of internalization, and after released into the cytosol, NPs and the encapsulated-pDNA interact with different molecules and organelles, which may trigger toxic effects and/or immune responses.

Different pathways are involved in inducing toxic effects in cells. The increase in ROS production, as an effect of interaction with the cellular membrane or after internalization, seems to be one of the main triggering mechanisms associated with other downstream effects [6,7,8,11]. Lysosomal escape is one of the critical mechanisms involved in a successful pDNA delivery, as the cargo needs to escape from lysosomal degradation to be released into the cytosol and enter the nucleus [10]. However, as the NPs escape from the lysosomes, toxic effects may occur as a result of the interaction of the lysosomal enzymes with different organelles and components in the cells. Other downstream effects of the administration of NPs are the stimulation of inflammatory markers, cell cycle arrest, apoptosis, necrosis as well as autophagy [12]. The imbalance in oxidation as well as the direct or indirect interaction between NPs and cellular organelles are the cause of the main types of toxic effects in these cells.

Delivering pDNA to the lungs shows many potential therapeutic benefits. The aim of this study is to assess the toxicological effects of the biodegradable polymer PLGA used to manufacture pDNA-encapsulated NPs in association or not with a novel CPP, on two lung epithelial cell lines (adenocarcinomic human alveolar basal epithelial cells (A549) and normal bronchial epithelial cells (Beas-2B cells)). CPP was used as an uptake enhancer. The physicochemical characteristics and particle internalization and release of these NPs was investigated. The potential toxic effects were assessed by measuring the mitochondrial activity, membrane integrity, oxidative stress, concentration of inflammatory markers and caspase-3 activation. Furthermore, the levels of apoptosis, necrosis and autophagy, and the effect on cell cycle was also investigated in both cell lines.

## 2. Materials and Methods

Poly(d,l-lactide–*co*–glycolide) (PLGA, Resomer RG502H, 50:50 copolymer ratio, inherent viscosity 0.16–0.24 dL/g and MW 7000–17,000) and the cell-penetrating peptide (CPP) were obtained from Evonik Industries, Dortmund, Germany. Ethyl acetate was purchased from J.T. Baker (Fisher Scientific, North Ryde, NSW, Australia), poly(-vinyl-alcohol) (PVA), Mowiol 4-88 from Sigma Aldrich Co. (Sigma, Castle Hill, NSW, Australia) and bovine serum albumin (BSA) from Cari (Cari Roth GmbH, Porirua, New Zealand). Calcium chloride and sodium hydrogen phosphate were obtained from Merck (Merck Chemicals Co., Bayswater, VIC, Australia). The cell-penetrating peptide (CPP), derived from lactoferrin and composed by 22 amino acids, was supplied by Evonik Nutrition and Care GmbH, Germany. All cell lines were acquired from ATCC. Beas-2B cells were used between passages 35–50, while A549 cells were used between passages 105–115.

Throughout this manuscript, all nanoparticles with pDNA-encapsulated has the suffix “-DNA” and for those formulated with CPP the suffix “-CPP” was included in the acronym.

### 2.1. Plasmid Purification and Extraction

A 5446 bp plasmid pc-DNA3-EGFP (Addgene plasmid # 13031—a gift from Doug Golenbock), encoding for EGFP was produced in *Escherichia coli* cells, amplified in lysogeny broth, following the protocol provided by Addgene. The plasmid was extracted and purified using a Qiagen purification kit (Cat. #12381), as per manufacturer’s instructions. The purity of the pDNA preparation was assessed using a Nanodrop spectrophotometer via 230/260 nm and 260/280 nm measurements. To confirm that the right plasmid was obtained, a restriction enzyme digest was performed. The pDNA was digested with two restriction enzymes, XbaI and XhoI (Sigma Aldrich), run on a 4% (*w*/*v*) agarose gel containing 2 µL SYBR^®^ Safe DNA Gel Stain, and electrophoresed at 100 V for 90 min in a Midi plus-1 Horizontal Electrophoresis System, (ME10-7-10, Major Science, Saratoga, CA, USA) coupled with BioRad Power Pac 200 (Bio Rad, CA, USA) electrophoresis power supply.

### 2.2. Nanoparticles Production

Nanoparticles were prepared using a double emulsion method (w_1_/o/w_2_) at 1:4:15 ratio. The organic phase was prepared as 2.4% of Resomer RG 502H in ethyl acetate. The first aqueous solution had BSA as a dispersing and protectant agent, while the second aqueous phase had PVA as a steric stabilizing reagent. The first emulsion was prepared by dispersing the aqueous solution (pDNA, 1.09 mM CaCl_2_, 1.3 mM Na_2_HPO_4_, and BSA 2% (*w*/*v*)) in ethyl acetate containing 2.4% PLGA (*w*/*v*). Dispersion was performed for 1.5 min in an ice bath at 18,000 rpm using an Ultra-Turrax^®^ (T18, IKA). The second emulsion was prepared by adding the primary emulsion into the second aqueous phase (1% PVA solution, *w*/*v*) and homogenizing for 3 min at 18,000 rpm. The homogenization was carried out in an ice bath as to protect pDNA structure from temperature-degradation. The organic phase was removed using a rotary evaporator. For flow cytometric and microscopy studies, Rhodamine B (Rhodamine B for fluorescence, Sigma Aldrich) at a concentration of 0.1% of polymer weight was added to the first aqueous phase. To remove any excess dye, after the NPs were prepared, samples were centrifuged at 13,200 rpm for 20 min at 4 °C. The supernatant was removed and the pellets resuspended at the desired concentration for further analysis. The addition of this novel CPP to the NPs occurred by incubating the freshly prepared NPs with an aqueous solution of the CPP to get a final 1:1.5 ratio of peptide: PLGA (mg:mg). Association between NPs and CPP occurred based on electrostatic interactions between these two components. The concentration of NPs in the assays was based on the PLGA concentration.

### 2.3. Nanoparticle Characterization

#### 2.3.1. Zetasizer and Zeta Potential Measurement

The zeta potential and size of the NPs, before and after addition of the CPP, were evaluated using a Malvern^®^ Nano Series ZS Instrument after 60 s of stabilization. Light scattering data were obtained at a detection angle of 173° and a temperature of 25 °C, and were subsequently analysed by the cumulant method to obtain the hydrodynamic diameters (nm) and polydispersity indices (μ/^2^) (PDI) of the NPs.

#### 2.3.2. Encapsulation Efficiency

The encapsulation efficiency was determined using an indirect method. Samples were centrifuged at 13,200 rpm for 30 min at 4 °C (Eppendorf Centrifuge 5415). The pDNA content was assessed in the supernatant using a Pico Green Kit (Thermo Fisher). Encapsulation efficiency (EE) was expressed as the percentage of pDNA found in the supernatant, compared to the total amount added to the first emulsion (Equation (1)).

(1) $$EE\;\left(\%\right)=\frac{Total\;amount\;of\;DNA\;-\;DNA\;in\;the\;supernatant}{Total\;amount\;of\;DNA}\times 100$$

### 2.4. Cell Culture

A549 cells (adenocarcinomic human alveolar basal epithelial cells) and Beas-2B (normal bronchial epithelial cells) were grown in Dulbecco’s Modified Eagle’s medium:F12 (Sigma, Sydney, NSW, Australia) containing 10% (*v*/*v*) fetal calf serum (Gibco, Sydney, NSW, Australia), 1% (*v*/*v*) non-essential amino acid solution (Sigma) with 1% (*v*/*v*) 2 mM L-glutamine solution (Sigma).

For NPs experiments, when using complete media (with 10% serum) 1% Penicillin/Streptomycin (Sigma) was added. For internalization studies, NPs were prepared in a solution of 25% Serum Free (SF) Media and 75% of NaCl 0.9% (*w*/*v*) (SF:NaCl). This solution was chosen as NaCl is the gold standard for nebulization. SF was added to the solution due to the acidity of NaCl (pH 5.5) in order to increase the pH to 6.8–7.0. Cells were grown in a humidified atmosphere at 37 °C, 5% CO_2_.

#### 2.4.1. Cell Toxicity

- **Mitochondrial Activity—MTS**: Cytotoxicity was determined in both cell lines using a MTS Cell Proliferation Assay Kit (Promega, Madison, WI, USA) following manufacturer’s instructions. This assay is based on the ability of viable cells to convert tetrazolium salt into a coloured formazan. Briefly, 5 × 10^4^ cells were seeded per well in a 96-well plate using complete media. After 24 h of incubation, treatments were added ranging from 0.075 to 1.2 mg/mL of PLGA, and incubated for 24 h. Both pDNA encapsulated nanoparticles and placebo (empty) nanoparticles were assessed. MTS reagent was added to the cells and incubated for 4 h, and samples read using a spectrophotometer (SpectraMax M2, Molecular Devices) at 492 nm. Cell viability for treated cells was determined in comparison to cells that had no treatment (media only).
- **Lactate Dehydrogenase Release—LDH:** The lactate dehydrogenase release assay (LDH assay) (RayBiotech) was used to determine the cytotoxic effect of the NPs by the quantification of plasma membrane damage. LDH is a stable enzyme found in the cytoplasm of all cells, and is quickly released to the cell culture supernatant when the cellular membrane is damaged. LDH assay was performed as per manufacturer’s instructions. Both cell lines (A549 and Beas-2B) were seeded (5 × 10^4^ cells/well) in a 96-well plate using complete media. After 24 h 100 µL of the treatment was added to the cells (pDNA-NPs from 0.1 to 1.2 mg/mL of PLGA prepared in SF:NaCl) and incubated for further 24 h. 100 µL of the supernatant of each well was transferred to another 96-well plate and 100 µL of the reaction mixture added to each well and incubated at room temperature for 30 min, protected for light. Samples were measured using a spectrophotometer at 450 nm. The controls in this assay included complete media (10% serum), SF:NaCl, NaCl 0.9%, Triton-X 2%, media with no cells and cells with no reagent added. LDH Release (%) was calculated in comparison to control cells with media only.

#### 2.4.2. Cellular Uptake

- **Cellular uptake by flow cytometry:** Cellular uptake of the NPs was assessed using flow cytometry BD Accuri^TM^ Flow Cytometer C6 (BD Accuri). Cells were seeded in 6-well plates (5 × 10^5^ cells/well) using complete media and allowed to attach and grow for 24 h. Cells were then incubated with the NPs–DNA and NPs–DNA–CPP, prepared in SF:NaCl for 3 h. Cells were washed twice with phosphate buffer solution (PBS), detached using trypsin and resuspended in PBS for flow analysis. Uptake was evaluated using the fluorescent channel 2 (FL2 detector, 585/625 nm; laser configuration of 3-blue 1-red, laser 488 nm). Measurements were carried out in triplicate and 10,000 events were acquired in the gated region of the forward-scatter/side scatter plot per sample. Cellular uptake results were calculated as the percentage of cells that internalized the particles (%) and the mean fluorescence intensity (a.u.). Internalization was assessed after 3, 24 and 48 h of incubation.
- **Cellular Uptake by Confocal Microscopy:** To confirm the internalization of NPs–DNA–CPP after 3 h, cells were visualized by confocal microscopy. Cells were seeded in 8 chamber-slides for fixed cell imaging (LabTek, Brendale, QLD, Australia) at a density of 50,000 cells per chamber for both cell lines. NPs treatments were added to the cells and incubated at 37 °C for 3 h for uptake studies. Cells were washed twice with warm PBS and fixed with 4% paraformaldehyde for 15 min. The cell membrane was counter-stained using Cell Mask Deep Red Membrane Stain (5 min incubation at 37 °C, Thermo Fisher) and the nucleus was stained using 4′,6-diamidino-2-phenylindole (DAPI) (1 µg/mL for 10 min). The chamber was removed and ProLong^®^ Gold Antifade Mountant (Thermo Fisher) was used to mount the coverslip.

Microscopy images were collected on Nikon A1R Confocal Fluorescence Microscope wit Apo LWD 40x WI lambdaS DIC N2 objective lens. Sequential scanning of 405 nm, 561 nm and 640 nm laser lines, was performed. DAPI was collected with 450/50 nm using a multialkili photomultiplier tube (PMT). Rhodamine B was collected with 595/50 nm emission using a GaAsP detector. And Deep Red Membrane dye was collected 700/50 nm emission using a PMT. Pinhole for all images was 0.6. Transmission detector was used for the bright field image. To assess cellular uptake a focal step (*Z* axis) of 0.25 µm thickness was used with the membrane stain as a reference for the cellular dimensions. Image processing occurred by deconvolution, using an iterative maximum likelihood algorithm (CMLE algorithm) implemented in Huygens Professional (Huygens, SVI, The Netherlands).

#### 2.4.3. Apoptosis and Necrosis

The effect of both NPs–DNA and NPs–DNA–CPP on the induction of apoptosis was evaluated using Annexin V-APC and Propidium Iodide by flow cytometry (BD Accuri^TM^ Flow Cytometer C6, BD). At first 3 × 10^5^ cells were seeded in 6-well plates and allowed to attach and grow for 24 h. Cells were treated with the NPs prepared in SF:NaCL for 24 h, followed by incubation in complete media (10% serum). Apoptosis and cell death were evaluated after 24, 48 and 72 h. Propidium Iodide (PI) staining was measured on channel 2 (FL2 detector; excitation at 585nm and emission at 625nm), while Annexin V-APC was measured in channel 4 (FL4 detector; excitation at 675 nm and emission at 700 nm), using Standard Filters (3 Blue 1 Red configuration). Cells were considered viable and non-apoptotic, when negative for both stains; considered necrosis, for those negative in Annexin V and positive for PI; in early apoptosis for those Annexin V positive and PI negative; and dead when positive for both stains. Hydrogen peroxide (3 mM) was used as a positive control. Experiments were performed in triplicate. For each treatment 10,000 events were acquired and results expressed as the percentage of total cells. Results were analysed using a two-way ANOVA (treatment × time), and means were compared using Tukey’s test.

#### 2.4.4. Cell Cycle

The cell cycle is regulated by a control system that is based on intracellular and extracellular signals. When exposed to high stress, this system may stop the cycle at one of the checkpoints, which is observed by the percentage of cells at each phase of the cell cycle: G0, G1, S, M, G2 [7]. The phases of the cell cycle can be differentiated according to the DNA content. On G0, the resting phase, the DNA content is at basal level and the same as G1, when the cell grows in size. During S phase the cell synthesizes DNA, while at G2 phase proteins are produced. The following phase is the mitosis when the two daughter-cells are formed [13]. Unregulated cycles may lead via different pathways to other downstream effects, such as inflammation and autophagy [2]. The effect of NPs on the cell cycle was assessed after 24 h of incubation using NPs formulated with or without CPP. Experiments were carried out as previously described for apoptosis. Cells were exposed to the NPs–DNA and NPs–DNA–CPP for 24 h. Treatments were then removed and cells were washed twice with PBS and detached. Cells were re-suspended in 70% cold ethanol and fixed for 30 min at 4 °C. Cells were then centrifuged (1000 rpm for 5 min at 4 °C), washed twice with PBS and stained with PI/RNase solution (BD) for 15 min in the dark. Cells were analysed by flow cytometry using fluorescent channel 2 (FL2), 10,000 events were recorder per sample. All experiments were performed in triplicate. Results were expressed as a percentage of the cells that were in each of the cell cycle phases (G0/G1, S, G2/M phase). The presence of sub-G1 population was also investigated, as it is also used as an indication of hypodiploid cells [14]. Means were analysed using two-way ANOVA (treatment × cell cycle phase) and compared using Tukey’s test to non-treated cells.

#### 2.4.5. Caspase-3

Caspase-mediated apoptosis is one of the pathways that NPs may induce in cells. Activation of caspase-3 is considered one of the essential steps for apoptosis, and has been widely used as a screening method for toxicity of NPs in different cell lines. The activity of caspase-3 was assessed using a colorimetric assay (ab39401, Abcam). Cells A549 and Beas-2B were seeded as described previously for the apoptosis assay. Cells were treated with NPs–DNA and NPs–DNA–CPP, prepared in 75:25 SF:NaCl. Vehicle and complete media containing 10% serum were used as controls. Caspase-3 activity was assessed after 24 and 48 h of incubation as per the manufacturer’s instructions. Briefly, cells were lysed using the buffer provided in the kit. After centrifugation at 10,000× *g* for 1 min, supernatant was collected for total protein determination and adjusted to 1 µg/µL of protein. Caspase-3 concentration in the samples were measured using caspase reaction mix provided in the kit with DEDV-p-NA substrate, in a clear flat bottom 96-well plate, and read in spectrophotometer at 400 nm. Caspase activity was corrected for total protein concentration (BCA Kit, Sigma). Results are expressed as a fold-increase in caspase-3 activity in comparison to non-treated cells. Results were analysed using two-way ANOVA (Treatment × time), means were compared using Tukey’s test.

#### 2.4.6. Nuclei Morphology by DAPI Staining

To visualize the effect of the NPs–DNA formulated with or without CPP on the morphology of the cellular chromatin, the cell nucleus was visualized using confocal microscopy. Cells were seeded in 8-well chamber-slides for fixed-cell imaging (LabTek) at a density of 50,000 cells per well for both cell lines. Cells exposed to NP–DNA–CPP for 6 h. After exposure, cells were fixed with paraformaldehyde 4% and stained with DAPI (1 µg/mL) for 10 min in the dark at room temperature. The chamber was removed and ProLong^®^ Gold Antifade Mountant (Thermo Fisher) used to mount the coverslip. Microscopy images were collected on Nikon A1R Confocal Fluorescence Microscope with Apo LWD 40x WI lambdaS DIC N2 objective lens. DAPI was collected with 450/50 nm using a multialkili photomultiplier tube (PMT). Pinhole for all images was 0.6. Transmission detector was used for the bright field image. For nuclei morphology images were analysed in Fiji Software as previously described [15] using the ratio between nuclei area and circularity. Three images were analysed with more than 50 nuclei analysed per cell line.

#### 2.4.7. Oxidative Stress by 2′,7′–dichlorofluorescin diacetate (DCFDA) dye

The reactive oxidative stress caused by exposing the cells to the NPs was assessed using DCFDA (2′,7′–dichlorofluorescin diacetate) dye. In this assay, DCFDA reacts with reactive oxygen species (ROS) to form 2′,7′–dichlorofluorescein (DCF), a fluorescent component, which is then measured by a spectrophotometer at 495/529 nm. Optimization of both dye concentrations and time points was performed prior (data not shown). Cells were seeded in a 96-well plate (50,000 cells/well) and allowed to attach and grow for 24 h. Media was then removed and cells were washed twice with PBS, before the addition of the DCFDA dye. For Beas-2B cells DCFDA dye was kept at 1 µM and incubation time was 15 min, while for A549 DCFDA concentration was 2 µM and incubation time 20 min. After incubation with the dye, cells were washed twice with PBS, followed by the addition of the treatments. ROS production was assessed after 15, 30 min, 1 h and 2 h of incubation. Hydrogen peroxide (0.03% *v*/*v*) was used as a positive control. Means were analysed using two-way ANOVA (Treatment × Time) and compared using Dunnet’s test to cells treated with vehicle only (NaCl: SF Media).

#### 2.4.8. Inflammatory Markers

The in vitro effect of the NPs formulated with or without CPP on inflammatory cytokines, including IL-6, IL-8 and tumor necrosis factor-α (TNF-α) was assessed using human ELISA kits (BD OptEIATM, San Jose, CA, USA) according to manufacturer’s instructions. The response of both A549 and Beas-2B cell lines was assessed after 24 h and 48 h of exposure with the NPs, in a similar procedure to the cellular uptake studies. The quantities of secreted IL-6, IL-8 and TNF-α in the test samples were determined using a standard curve measured with purified recombinant human IL-6, IL-8 and TNF-α provided in the kit, with a limit of detection of 1.56, 2.34 and 7.81 pg/mL, respectively. Results are presented as fold difference to the non-exposed cells. Lipopolysaccharide (LPS) and saline were used as controls.

#### 2.4.9. Autophagy

The effect of NPs, formulated with or without CPP, on inducing autophagy was assessed using Autophagy Assay Kit (Sigma, Castle Hill, NSW, Australia), as per manufacturer’s instructions. Briefly, cells (A549 and Beas-2B) were seeded using complete media in a black clear bottom 96-well plate (Corning, Sigma) at 2 × 10^4^ cells/well and allowed to attach and grow for 24 h. Cells were exposed to the NPs, formulated with or without CPP for 24 h. Treatments were removed and the supplied autophagosome reagent was added to each well and incubated for 1h at 37 °C, 5% CO_2_. Cells were carefully washed 4 times with washing buffer provided in the kit and fluorescence signal measured at 360/520 nm using a spectrophotometer. NPs–DNA formulated or not with CPP were tested in a concentration range from 0.4 to 2.4 mg/mL of PLGA. CPP alone was also tested at different concentrations. Results are presented in relation to the media control signal and were analysed using one-way ANOVA, means were compared using Tukey’s test.

### 2.5. Statistical Analysis

Results are expressed as mean ± Standard deviation (StDev). All experiments were conducted in triplicate. Statistical analysis was performed using one-way analysis of variance (ANOVA) and compared by Dunnett’s test, unless otherwise stated. Differences were considered significant when * *p* < 0.05; ** *p* < 0.01; *** *p* < 0.001; **** *p* < 0.0001.

## 3. Results

### 3.1. Nanoparticles Production and Characterization

**Figure 1:**
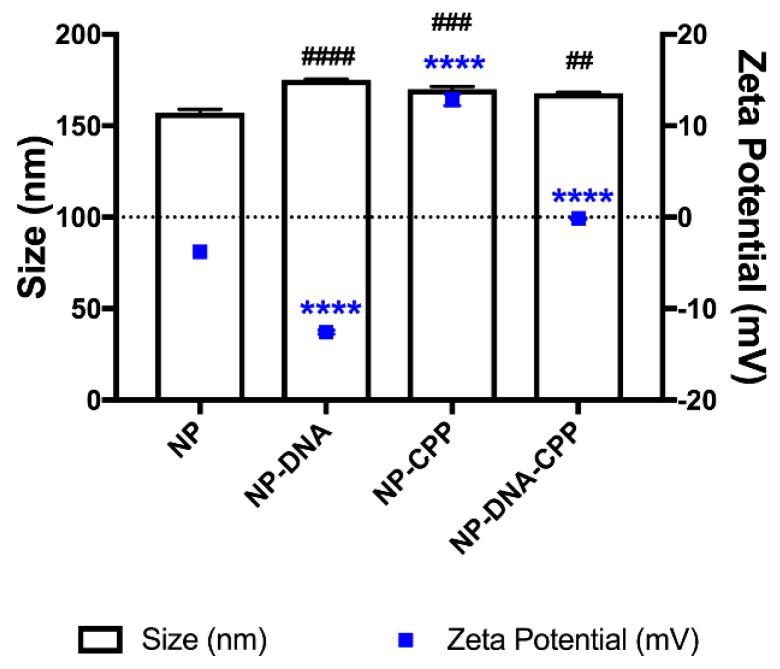
Particle size (columns) and zeta potential (dots) of nanoparticles (NPs) coated with or without cell-penetrating peptides (CPPs), before and after pDNA encapsulation. (*n* = 3 ± StDev; Statistical difference in size is shown in hash (#) and for zeta potential in stars (*). **** *p* < 0.0001; ## *p* < 0.01; ### *p* < 0.001; #### *p* < 0.0001).

Nanoparticles were produced using a double-emulsion technique. Particles smaller than 200 nm in size were considered suitable for intracellular delivery (Figure 1). The surface charge increased with the addition of CPP (*p* < 0.0001). Encapsulation of pDNA was successful using this technique, with 96.7 ± 0.4% of pDNA encapsulated in the NPs–DNA. Encapsulation of pDNA and addition of CPP increased the particle size of the NPs, specifically for NP–DNA (*p* = 0.0001), NP–CPP (*p* = 0.0004) and NP–DNA–CPP (*p* = 0.0015), respectively, in comparison to placebo NPs. Particle size distribution of all particles, before and after addition of CPP, was narrow with PDI smaller than 0.1.

### 3.2. Cell Toxicity

The toxicity of the NPs–DNA was assessed using MTS assay, which evaluates the mitochondrial respiratory system to determine a non-toxic concentration to be used in this study. NPs-DNA were assessed using a range of concentrations from 0.075 to 1.2 mg/mL. NPs were considered toxic when viability was lower than 80% (Figure S1, Supplementary Materials). For both NP–DNA–CPP and NPs-DNA, as well as CPP alone, no toxic effects were observed in either A549 and Beas-2B cells for all concentrations tested.

The effect of the NPs on the membrane integrity was assessed using LDH assay. Due to the ability of CPP to enhance internalization, membrane integrity was assessed to ensure that NPs–CPP did not occur by damaging the cellular membrane. LDH leakage was lower than 20% at all concentrations tested, and NPs–DNA and NPs–DNA–CPP were considered non-toxic in both cell lines (Figure S2, Supplementary Materials).

For the following studies the concentration of NPs–DNA was kept as 0.4 mg/mL of PLGA as this concentration showed to be non-toxic in all cell lines tested.

### 3.3. Cellular Uptake

**Figure 2:**
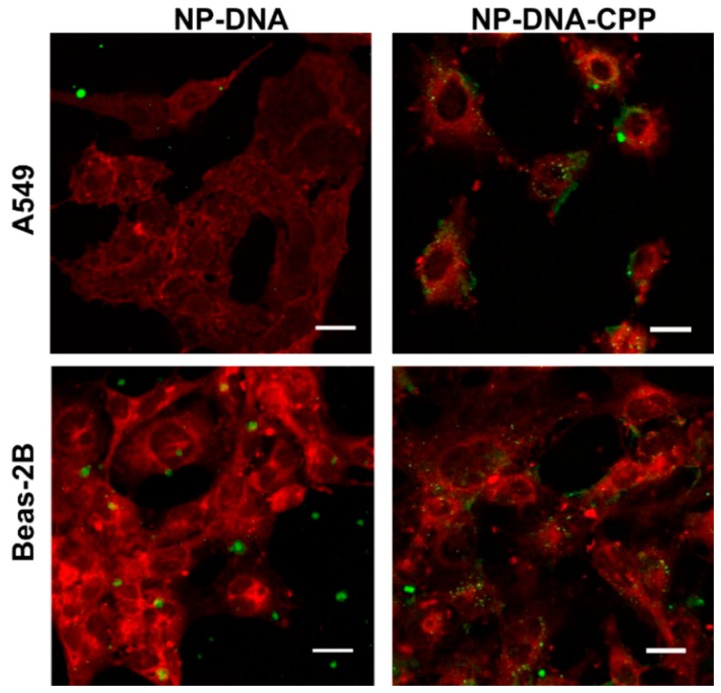
Confocal microscopy image of the cellular uptake of both NP–DNA and NP–DNA–CPP in both A549 and Beas-2B cells. (Red) Cellular membrane; (Green) NP–DNA and NP–DNA–CPP. Error bar indicates 20 µm.

The effect of the addition of CPP on enhancing cellular uptake was assessed in both A549 and Beas-2B cells after incubation for 3 h with the NPs–DNA (formulated with or without CPP). When assessed by flow cytometry, a significant increase (*p* < 0.0001) in the percentage of cells was observed for cells treated with NPs–DNA–CPP, in comparison to NP–DNA (Table 1), indicating that the addition of CPP to the nanoparticles was essential for internalization. Internalization was confirmed using confocal microscopy (Figure 2), by overlapping the images from the cellular membrane (red) and the labelled NPs (green) in a z-stack imaging, corroborating the flow cytometer results.

### 3.4. Apoptosis

**Figure 3:**
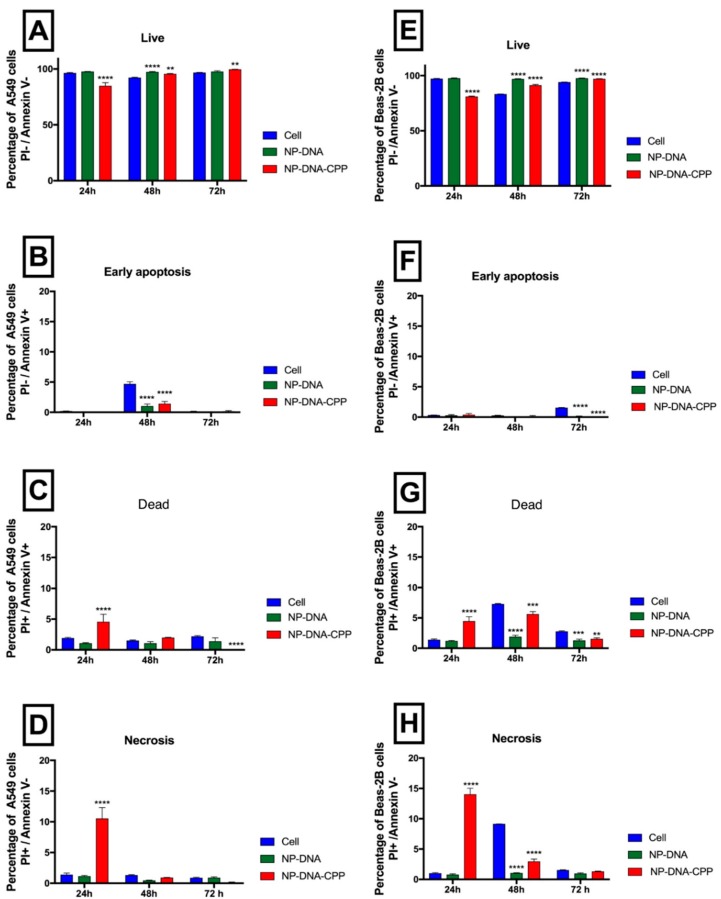
Apoptosis after 24, 48 and 72 h of A549 cells (left column) and Beas-2B cells (right column) when exposed to NPs coated with (red) or without (green) CPP and analysed by flow cytometry. Cells were classified into four different stages according to both Annexin V and PI dyes: live (**A**,**E**), early apoptosis (**B**,**F**), late apoptosis (**C**,**G**) and necrosis (**D**,**H**). All treatments were compared to cell media control (blue) using two-way ANOVA and Tukey’s Test. (*n* = 3; ** *p* < 0.01; *** *p* < 0.001; **** *p* < 0.0001).

Apoptosis has been widely associated with toxic effects often induced by NPs. The ability of NP–DNA formulated with or without CPP to induce apoptosis was assessed after 24, 48 and 72 h, in both A549 (Figure 3A–D) and Beas-2B cells (Figure 3E–H). One of the early signs of apoptosis is the appearance of phosphatidylserine residues on the outer plasma membrane. This process can be detected by using Annexin V dye, which when associated with propidium iodide, a dye that stains nucleic acids when the cellular membrane integrity had been damaged, gives an indication of apoptotic cell death.

Cells were considered to be in the early stages of the apoptotic phase, when positive only for Annexin V, due to the binding of Annexin-V dye to Phosphatydilserine on the outer leaflet of the membrane. In both A549 cells (Figure 3B) and Beas-2B (Figure 3F) cells, less than 2% of the cells were found in early apoptosis at all time points evaluated. When cells were positive for both dyes (Annexin V and PI) cells were considered to be dead. A significant increase in the percentage of dead cells in A549 cells (*p* < 0.0001) was observed for NPs–DNA–CPP treated cells in comparison to both NPs–DNA and cell control at 24 h (Figure 3C). No significant difference (*p* > 0.05) was observed at 48 h, while at 72 the percentage of dead cells exposed to NPs–DNA–CPP in A549 was 0.03 ± 0.026% (Dot plots in Figure S3, Supplementary Materials). In the case of the Beas-2B cell line, although a significant increase (*p* < 0.0001) in dead cells at 24 h was observed, when assessed at 48 h the percentage of cells in this phase significantly decreased (*p* < 0.01) in comparison to cell media. For necrotic cells, identified for PI positive only, a significant increase is observed at 24 h for both cell lines (*p* < 0.0001), which is not observed at both 48 and 72 h (Dot plots in Figure S4, Supplementary Materials).

### 3.5. Caspase-3

**Figure 4:**
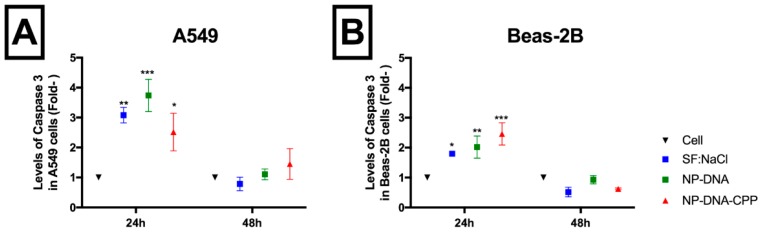
Caspase-3 level as a fold-change to the exposure to NPs coated with (red) or without (green) CPP, SF:NaCl (blue) in comparison to cell control (black) in both (**A**) A549 cell and (**B**) Beas-2B, after 24 and 48 h (*n* =3 ± Stdev); Means were analysed using two-way ANOVA and compared using Tukey’s test.* *p* < 0.05; ** *p* < 0.01; *** *p* < 001.

Caspase-3 activity was detected using a colorimetric assay and it is represented as the fold activity in relation to the media control (with 10% serum) (Figure 4). In A549, a significant increase in caspase-3 activity was observed for NPs–DNA–CPP (*p* = 0.03) and NPs–DNA (*p* = 0.0001) treatments in comparison to cell media (containing 10% serum) following a 24-h incubation (Caspase 3 of 0.023 ± 0.003). When NPs–DNA–CPP was compared to SF:NaCl treatment (vehicle), caspase-3 activity level was similar (*p* > 0.05).

For Beas-2B, a significant increase was observed in caspase-3 activity for NPs–DNA–CPP (*p* < 0.0001) and NPs–DNA (*p* = 0.0015) treatments in comparison to cell media (0.032 ± 0.004). When compared to SF:NaCl, NPs–DNA–CPP showed a modest increase (*p* = 0.0493) of caspase-3 activity. When assessed at 48 h, no significant difference was observed for either NPs–DNA treatments (*p* > 0.05) in both cell lines.

### 3.6. Cell Cycle

Cell cycle arrest has been observed as an effect of NPs exposure. This cell cycle assay was determined based on DNA staining, in which G2/M presents doubles the content of G0/G1. Gating strategy is detailed on Supplementary Material (Figures S5 and S6, Supplementary Materials). When the DNA content is lower than those observed in the G0/G1, there is an indication of either apoptotic or necrotic cells, as the DNA is getting damaged or fragmented. Cell cycle arrest is correlated to the stress caused by the NPs that may lead to the cells to stop at one of the phases of the cycle. In this study, when the percentage of Beas-2B cells in each phase was compared to the SF:NaCl vehicle, in which the NPs were prepared. NPs–DNA showed significant differences in both G0/G1 and G2/M phase (Table 2). When SF:NaCl was compared to the media control (with 10% serum) a significant decrease in G2/M population was observed along with an increase in G0/G1, mostly due to the serum starvation which induce cell arrest in G0. Interestingly, although NPs–DNA–CPP were prepared in SF:NaCl, the percentage of cells in G0/G1 was significantly lower (*p* < 0.0001) in NPs–DNA–CPP. For A549 cells, serum starvation in both NPs and SF:NaCl seems to induce cell cycle arrest in G0/G1, with a significant decrease of cells in S phase (*p* < 0.0001).

However, when compared to cell with complete media treatment, no statistical differences were observed (*p* > 0.05). In both cell lines when NPs–DNA were formulated with CPP (NPs–DNA–CPP) there was a significant increase (*p* < 0.0001 for Beas-2B and *p* < 0.01 for A549 cells) in the population in Sub-G1 phase, which is a marker for hypodiploid cells.

### 3.7. Oxidative Stress

**Figure 5:**
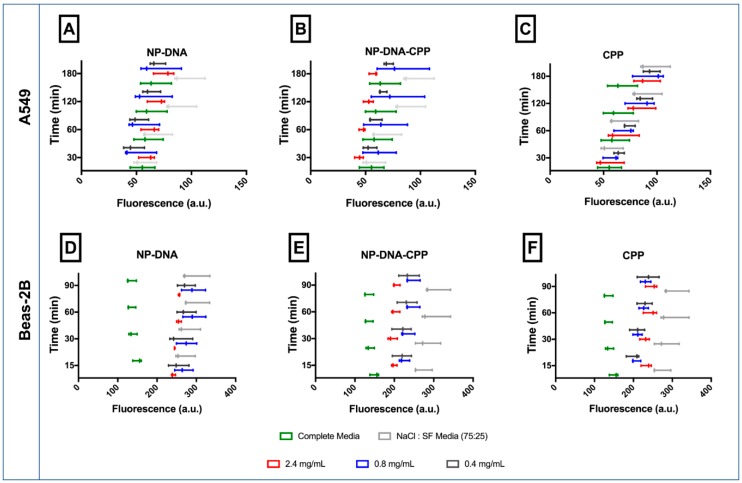
Oxidative stress of both A549 (**A**–**D**) and Beas-2B (**E**–**H**) cells after being exposed to NP–DNA (**B**,**D**), NP–DNA–CPP (**C**,**G**) and CPP alone (**D**,**H**). (*n* = 3 ± StDev). Means were statistically analysed by two-way ANOVA and compared against SF:NaCl per phase amongst treatments using Tukey’s test.

Nanoparticles commonly cause oxidative stress in cells. This effect was assessed over 90 min in Beas-2B cells and over 180 min for A549 cells (Figure 5). No significant effect (*p* > 0.05) was observed in inducing oxidative stress in A549, with NPs–DNA or CPP alone. After 120 min, a decrease in ROS was observed for NP–DNA–CPP cells (*p* < 0.05). On the contrary, Beas-2B oxidative status was significantly affected by both NPs–DNA (formulated with or without CPP). When incubated with NPs–DNA (without CPP) the oxidative stress was similar to those observed for SF:NaCl at all time points evaluated (*p* < 0.05). However, when NPs–DNA–CPP was added, a significant decrease in the oxidative stress was observed in comparison to SF:NaCl from 30 min of incubation (*p* < 0.05), similarly to the results from CPP alone (*p* < 0.05). The reduction in oxidative stress observed for NPs–DNA–CPP or CPP alone was not concentration related (*p* > 0.05).

### 3.8. DNA Damage via Nuclei Morphology

One of the potential problems of NPs delivery is damaging the cellular DNA, since NPs can directly interact with the DNA or induce excessive stress. To assess the effect of the NPs–DNA on the cell nuclei, cells were stained with DAPI and visualized microscopically after exposure. The images were analysed in order to obtain two parameters: nuclei area and circularity [15]. The ratio between these two parameters is associated to the nuclear morphology, and extrapolated to DNA damage [15]. For each treatment, the morphology of more than 50 nuclei was analysed (Figure S7A, Supplementary Materials). Results of Beas-2B nuclei morphology, analysed the ratio between nuclei area and circularity parameters, showed no significant difference (*p* > 0.05) after exposure to both NPs–DNA formulated with or without CPP, while in A549 NPs–DNA significant decrease (*p* < 0.05) area/circularity ratio and NPs–DNA–CPP increased (*p* < 0.01) area/circularity ratio. Images of nuclei in both cell lines can be seen in Supplementary Figure S7B,C, Supplementary Materials.

### 3.9. Inflammatory Markers

**Figure 6:**
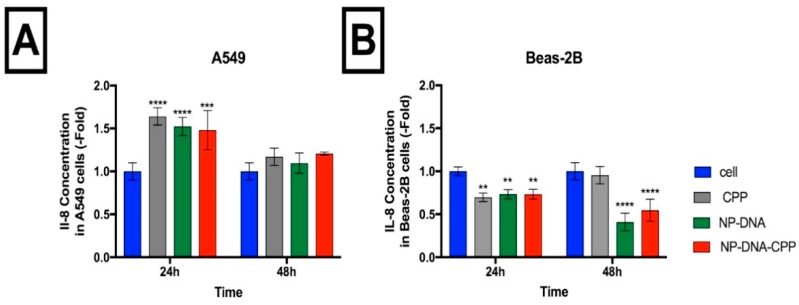
Effect on the pro-inflammatory cytokine IL-8 after 24 and 48 h of exposure to NPs coated (NP–CPP) or without (NP), and CPP alone for both: (**A**) A549 cells, and (**B**) Beas-2B cells. All treatments were compared to the non-exposed cell control (blue) using 2-way ANOVA. (*n* = 3; ** *p* < 0.01; *** *p* < 0.001; *** *p* < 0.0001) followed by Tukey’s Test.

The effect of the NPs–DNA on the production of inflammatory markers was assessed over 24 and 48 h (Figure 6). For the Beas-2B cell line, when compared to basal levels, NPs-DNA, NPs–DNA–CPP and CPP alone, significantly (*p* < 0.01) reduced IL-8 after 24 h. This decrease in pro-inflammatory cytokines was maintained at 48 h.

When A549 cells were exposed to the NPs-DNA, NPs–DNA–CPP and CPP alone, a significant (*p* < 0.001) increase was detected in IL-8 levels. This increase was not sustained at 48 h, with no significant (*p* > 0.05) difference observed between the treatments and the basal cell level.

The levels of both IL-6 and TNF-α in the samples was lower than the limit of detection of the assay.

### 3.10. Autophagy

**Figure 7:**
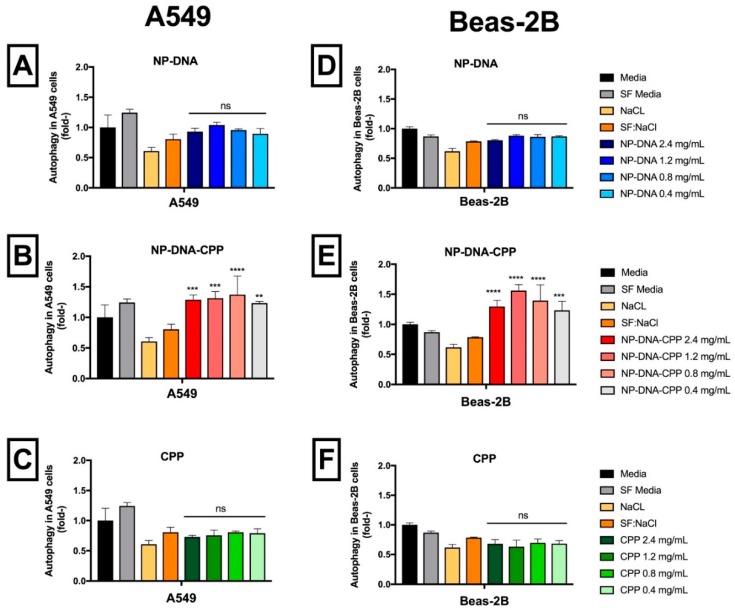
Autophagy in A549 (**A**–**C**) and Beas-2B (**D**–**F**) cell lines after exposed to NP–DNA (**A**,**D**), NP–DNA–CPP (**B**,**E**) and CPP alone (**C**,**F**). All treatments were compared to the SF:NaCl control using one-way ANOVA, followed by Tukey’s Test. (*n* = 3; ns: not significant, ** *p* < 0.01; *** *p* < 0.001; *** *p* < 0.0001).

Autophagy was assessed using both NPs–DNA formulated with or without CPP. When compared to the vehicle SF:NaCl, NPs-DNA treatments did not significantly (*p* > 0.05) induce autophagy in either A549 or Beas-2B cell lines (Figure 7). In cells treated with NPs–DNA–CPP, autophagy was significantly increased (*p* < 0.001) at all concentrations tested, for both cell lines. However, this increase was not significantly different (*p* > 0.05) from the levels observed in the complete media treatment for A549 at all concentrations tested, and in Beas-2B at 0.4 mg/mL of PLGA.

## 4. Discussion

In this study, pDNA-encapsulated PLGA nanoparticles were formulated with (NPs–DNA–CPP) or without (NPs–DNA) the addition of CPP. The effect of these two types of NPs–DNA on the ability to encapsulate and enter two lung epithelial cell lines was compared. The cell lines A549 (alveoli adenocarcinoma derived) and Beas-2B (bronchial healthy derived) were chosen, as they represent suitable alveoli and bronchial models, respectively. The use of both cell lines allowed for the investigation of the potential harm of NPs on the whole lung region. Following efficient internalization of NPs–CPP, the toxic effect of these particles was assessed by metabolic assay, membrane integrity, apoptosis, caspase-3, DNA damage, inflammatory markers, oxidative stress and autophagy.

### 4.1. Nanoparticle Characterization and Cytotoxicity

The size and shape of NPs have been associated to different cytotoxic effects, with spherical particles, as those produced in this study showing little or no toxicity in comparison to needle- or rod-shaped particles [16]. Numerous shapes of NPs have been produced to increase internalization and induce endosomal escape [5,16,17], an essential step for an efficient pDNA delivery. Cytotoxic effect has been associated to NPs in a size-dependent manner, most likely due to the high surface-to-volume ratio [5,6,18] that increases the reactivity of these molecules in the cell. When cytotoxicity was assessed using a metabolic activity assay (MTS; Figure S1, Supplementary Materials) from 0.075 to 1.2 mg/mL cells were more than 70% viable at all concentration for both treatments. Different results were observed by Grabowski et al., where neutral PLGA/PVA nanoparticles at concentrations higher than 1 mg/mL were cytotoxic (less than 75% viability) in A549 cells, when assessed in a different metabolic assay (MTT assay) [19]. These differences in cytotoxicity may be related to both size and physicochemical characteristics of the NPs, as their manufacturing process was different.

For membrane integrity (Figure S2, Supplementary Materials), no toxic effects were observed from concentrations of 0.1 to 1.2 mg/mL in both Beas-2B and A549 cells, respectively. These results were corroborated by the lack of effect on membrane integrity also observed for PGLA/PVA NPs [19] at both 0.1 and 1 mg/mL in A549 cells. Spherical-shaped PLGA–PEG NPs have also shown no effect on LDH release from 25 to 250 µg/mL [16].

Toxic effects seem to be directly correlated to the concentration of NPs to which the cells are exposed [18]. In this study, when both cell lines were exposed to 400 µg/mL of NPs, no toxic effects were observed in either cell lines, for both NP–DNA formulated with or without CPP. These results agree with those obtained by Xiong et al., that showed that PLGA NPs larger than 100 nm [18], similar to those used in this study, did not trigger any toxic effects up to 300 µg/mL. Platel et al., also showed non-toxic effects associated to negatively charged PLGA NPs up to 500 µg/mL in a human bronchial epithelial cell line (16HBE14o-) [20].

The surface charge of the NPs is also associated to potential toxic effects, with cationic NPs showing acute cytotoxicity that also trigger inflammation [17,20]. The increase in surface charge is a common strategy used to enhance NPs internalization [21], and showed to be essential for an efficient intracellular delivery (Table 2; Figure 3). In this study, adding CPP to the NPs, led to an increase in surface charge to a neutral zeta potential, with no further effect on both MTS and LDH assays, corroborating the results from Platel et al. in which neutral and negatively charged NPs did not influence cell viability [20].

### 4.2. ROS Production, Inflammatory markers, Apoptosis, Nuclei Morphology and Autophagy

One of the key mechanisms of nanotoxicity is the generation of ROS within the cells. The decrease in particle size and the higher surface area of NPs, increases the reactivity between the NPs and the cells and, consequently, ROS production. When the accumulation of ROS exceeds the antioxidant capacity of the cell, oxidative stress is induced, which may lead to damage of DNA and proteins, unregulated cell signalling, inflammation, apoptosis and even carcinogenesis [6,8,11].

While no oxidative stress was observed for either NPs–DNA in A549 cells and Beas-2B cell line, when CPP was added to the nanoparticles (NPs–DNA–CPP) a significant decrease in ROS production was observed for both cell lines. In Beas-2B CPP alone was also able to significantly reduce ROS production, indicating that the decrease in oxidation observed in cells exposed to NP-DNA-CPP may be related to the presence of the CPP on the surface of the nanoparticles. Although a direct correlation has been made in literature to support the relationship between particle surface area and generation of ROS [6,20,22], our results support the hypothesis that this correlation can be affected by surface coating. Surface modifications have been shown to modify NPs properties [20,23] in relation to both particle surface charge and coating. For instance, positively charged PLGA NPs have been shown to increase ROS production in 16HBE14o-cells, while negatively charged PLGA NPs did not induce oxidative stress in the same cell line [20].

The ability to induce ROS generation has been correlated to the NPs internalization. Platel et al. showed that positively charged NPs did not induce ROS production when endocytosis was inhibited [20]. In this study, negatively charged particles (NPs–DNA) showed very little internalization efficiency (2.53 ± 0.52%) in Beas-2B cell, although it was able to induce a significant increase in ROS production, indicating that either the NPs–DNA induced ROS generation from interacting with the cell before internalization, or the small percentage of internalized NPs–DNA was able to induce a very strong oxidative stress. Interestingly, adding CPP to the NPs–DNA, which showed 83.85 ± 1.211% of internalization efficiency, reduced the oxidative stress induced by the vehicle SF:NaCl in the same cell line.

Other toxicological outcomes, such as inflammation, have also been associated with the intracellular delivery of NPs. When harmful particles are inhaled, the activation of receptors (toll-like receptors and NOD-like receptors) start a downstream signalling pathway that can lead to the production of different cytokines and activation of an immune response [2,12]. Evidence has shown that biodegradable NPs are able to induce a lower inflammation response both in vitro and in vivo, when compared to non-biodegradable NPs of similar size and surface area [22]. The increase in ROS generation was reported to be an important mediator of IL-8 and IL-1β signalling pathways [24], mostly due to the activation of toll-like receptor 4 [25], expressed in both of cell lines studied in this study [26,27]. ROS can also trigger NF-kB signalling, increasing IL-1 and IL-18, inducing the activation of inflammasomes leading to an inflammation status [25]. In this study, although the levels of IL-8 increased in A549 cells after 24 h treatment for both NPs–DNA and NPs–DNA–CPP, this increase was not ROS-induced, as the concentration of ROS did not significantly changed for this cell line. Furthermore, although a decrease in IL-8 was observed in Beas-2B after 24 and 48 h after exposure to NPs and NPs–DNA–CPP, different effects were observed in the ROS production when NPs had CPP added (Figure 5 and Figure 6), corroborating the hypothesis that stimulation of IL-8 pathway by the NPs–DNA was not correlated to ROS production in both cell lines. The different responses observed for the cell lines in this study may be related to the activation/inhibition of different pathways or downstream signalling, and should be futher investigated.

An important aspect of gene delivery is related to the stimulation of a lysosome-escape mechanism by the NPs. Most of the NPs, as well as the NPs–DNA–CPP from this study, enter the cells via endocytosis, in which the endocytic vesicles will maturate into the lysosomal compartment, where the cargoes get degraded. To achieve an efficient pDNA delivery, the NPs need to go through an endosomal-escape mechanism, which is mostly achieved by the proton-sponge effect. This lysosomal release comprises of a bursting mechanism to release the cargoes into the cytosol, along with the lysosomal hydrolases that may further induce inflammation [12]. Although the results of this study suggest that the NPs–DNA–CPP produced may efficiently escape from the lysosomes, in both A549 and Beas-2B cell lines, no strong inflammation effect occurred.

Lysosomal content release was also associated with an increase in DNA damage and apoptosis, mostly due to the digestion of essential proteins, triggering DNA fragmentation and apoptosis signalling [16]. Inducing apoptosis is a quite common toxic effect observed after administration of NPs. Several types of stress can lead to apoptosis after NPs exposure, such as the increase in oxidative stress, lysosomal and mitochondrial dysfunction, endosomal reticulum stress or autophagy dysfunction [12,28]. In this study, less than 5% of both cells were in an early apoptotic state. Although a significant increase was observed at 24 h for dead cells, the percentage was lower than 6%. Further incubation did not show significant differences between NPs–DNA and NPs–DNA–CPP and the control, probably due to cell proliferation. When these results were compared to those obtained from the caspase-3 assay, which activation is critical for the initiation of apoptosis, no significant difference was observed from both NPs-treated cells and the vehicle SF:NaCl (Figure 4). Interestingly the levels of caspase 3 in both NPs and vehicle was higher in both cell lines after 24 h of incubation, in comparison to the control with complete media (10% serum). This increase may be related to the fact that the vehicle consisted of SF media and NaCl, and serum starvation has been associated with apoptosis via caspase 3 activation [29]. At 48 h, the levels of caspase-3 decreased to similar levels than those observed in the control cell with complete media (10% serum). This result is explained by the fact that in our experiment, after 24 h complete media was added to all wells in the assay, restoring primary functions and reversing starvation. Among all caspases, caspase 3 is one of the executer caspases, which gets cleaved to initiate the apoptosis cascade, however other caspase-mediated pathways may be involved in inducing apoptosis and should be further investigated.

When cell death induced by necrosis was quantified, after 24 h of incubation NPs–DNA–CPP induced necrosis. Specifically, 10.5 ± 1.7% for A549 cells, and 14.1 ± 1.0% for Beas-2B, respectively. NPs–DNA did not induce necrosis in either cell lines assessed. These results agree with those obtained for the detection of autophagy in these cell lines, where results showed that cells treated with NPs–DNA–CPP presented a significant increase in autophagy in comparison to non-treated cells. This effect can be correlated to the lysosomal-escape mechanism that occurs in NPs–DNA–CPP in both cell lines, that induce release on lysosome contents into the cytosol, causing acidification of cytosolic pH with consequential necrosis [12,28]. Cationic NPs were shown to induce necrotic cell death as a response to the strong stimulus caused by the NPs [28]. This stimulus also seems to be correlated to NPs concentration to which the cells are exposed [28]. It is noteworthy that CPP alone was not able to induce autophagy, corroborating the hypothesis that NPs–DNA–CPP induces autophagy is some of the cells after internalization and it is not due to an external stress.

Changes in nuclear morphology was assessed using DAPI staining. As shown in Supplementary Figure S4, NPs–DNA and NPs–DNA–CPP did not affect the nuclei morphology in Beas-2B cell line. In A549 the changes in nuclear area/circularity ratio observed in both NPs–DNA may be associated to apoptotic or necrotic cells [15]. When circularity was analysed alone, no differences were observed (data not shown) indicating that the changes in nuclear area/circularity ratio were mostly related to the nuclear area than changes in the circularity. The increase in nuclear area/circularity ratio, occurred in NP–DNA–CPP may be related to the presence of necrotic cells, as nuclei swelling is one of the characteristics of this type of cell death.

Other forms of cellular damage can also be detected by assessing the effect of NPs–DNA on the cell cycle, as the cell in response to different stimuli will control the progress of the cell through the cell cycle phases. The major changes observed in A549 cells (increase in G0/G1 and decrease in S phase) when exposed to both cell NPs–DNA are likely to be related to serum starvation, in which the cells decrease their metabolic activity (decrease in S phase), remaining in the resting phase (increase in G0). The same results were observed in Beas-2B with increase in G0/G1 and decrease in G2/M phase. When compared to cell media (10% serum) the decrease in G2/M phase was not significant for NP-DNA treated cell in Beas-2B, this results may be correlated to the degradation of the PLGA polymer into poly lactic acid (PLA) and poly glycolic acid (PGA) [4], compounds that can enter the Krebs cycle to provide ATP. The effect of the NPs–DNA on DNA fragmentation can also be detected via cell cycle by visualisation of a sub-G1 population in the flow cytometric plot. In both cell lines an increase in sub-G1 population was observed for NPs–DNA–CPP (Table 2), mostly due to the increase of necrotic cells at 24 h, as observed in Figure 1.

In summary, cells exposed to the NPs–CPP showed low inflammatory response, low levels of apoptosis and no activation of caspase-3. An increase in necrotic cells (between 10%–15%) is observed after 24 h of incubation with NPs–DNA–CPP, which may be related to the lysosomal-escape mechanism that the particles induce after internalization. An increase in sub-G1 population (less than 10%) and the changes in DNA morphology occur by exposing the cells to NPs–DNA–CPP, mostly due to the presence of necrotic cells. Autophagy appears to be induced by NPs–DNA–CPP in both cell lines, and this seems to also be correlated to the lysosomal escape mechanism. Although oxidative stress is one of the main toxic mechanisms observed for NPs, in this study NPs–CPP showed decreased ROS production on Beas-2B cells and no effect on A549 cells, potentially indicating an antioxidant effect of this CPP on Beas-2B, a healthy-derived bronchial epithelial cell.

## 5. Conclusions

In this study, the toxic effects of DNA-encapsulated PLGA nanoparticles formulated with or without CPP on two lung epithelial cell lines were investigated. Results show that NPs–DNA–CPP did not induce apoptosis, oxidative stress or cell cycle arrest on either cell, with a minor increase in necrotic cells and autophagy. Therefore, this system appears to be safe to be delivered to lung epithelial cells. Further investigations should be undertaken to assess the safety of these nanoparticles in other lung related systems and better understand the effect of these particles on necrosis and autophagy.

## Figures and Tables

**Table 1:** Cellular uptake of NPs–DNA coated with or without CPP in both adenocarcinomic human alveolar basal epithelial cells (A549) and normal bronchial epithelial cells (Beas-2B cells) (*n* = 3 ± StDev).

Treatment	A549 Cells (%)	Beas-2B Cells (%)
Cell Control	0.94 ± 0.16	0.95 ± 0.03
CPP	1.34 ± 0.52	0.85 ± 0.25
NPs	4.20 ± 0.82 ^a^	2.53 ± 0.52 ^b^
NPs-CPP	96.76 ± 1.71 ^c^	83.85 ± 1.21 ^c^

Means (*n* =3 ± StDev) of each cell line were statistically analysed by one-way ANOVA and compared each treatment with cell control using Tukey’s test. Superscript letter indicates statistical difference: ^a^ *p* < 0.05; ^b^ *p* < 0.01; ^c^ *p* < 0.0001. NPs and NPs–CPP were labelled with Rhodamine B at 0.1% of polymer weight.

**Table 2:** Percentage of Beas-2B and A549 cells in each cell cycle phase (G0/G1, S, G2/M) analysed using propidium iodide by flow cytometry after exposure to NPs–DNA coated with or without CPP.

Cell Cycle Phases	Beas-2B			
	Cell	SF:NaCl	NP-DNA	NP-DNA-CPP
G0/G1	51.53 ± 3.34 ^c^	58.48 ± 3.08	54.46 ± 1.33 ^a^	49.96 ± 1.53 ^d^
S	11.81 ± 1.00	9.115 ± 1.76	10.48 ±1.19	11.00 ± 1.57
G2/M	35.74 ± 0.51 ^b^	30.28 ± 1.37	34.70 ± 1.03 ^a^	30.99 ± 0.85
Sub-G1	0.95 ± 0.07	2.02 ± 0.11	1.02 ± 0.03	8.27 ± 0.26 ^d^
	**A549**			
	**Cell**	**SF:NaCl**	**NP-DNA**	**NP-DNA-CPP**
G0/G1	62.06 ± 1.41 ^d^	77.83 ± 0.05	74.88 ± 0.89	71.08 ± 1.30 ^c^
S	20.20 ± 3.01 ^d^	6.79 ± 0.25	6.52 ± 2.55	5.87 ± 0.66
G2/M	16.54 ± 4.02	14.61 ± 1.08	18.28 ± 2.31	16.33 ± 1.65
Sub-G1	0.76 ± 0.40	0.70 ± 0.11	0.47 ± 0.04	7.09 ± 0.39 ^b^

Means (*n* = 3 ± StDev) were statistically analysed by two-way ANOVA and compared against SF:NaCl per phase amongst treatments using Tukey’s test. Superscript letter indicates statistical difference: ^a^ *p* < 0.05; ^b^ *p* < 0.01; ^c^ *p* < 0.001; ^d^ *p* < 0.0001.

## Supplementary Materials

The following are available online at http://www.mdpi.com/1999-4923/11/1/12/s1, Figure S1: Cytotoxicity of NP-DNA and NP-DNA-CPP in (left panel) A549 and (right panel) Beas-2B cells, Figure S2: Membrane integrity, via LDH Leakage assay, of both Beas-2B (circles) and A549 cells (squares) exposed to (A) NPs or (B) NPs-CPP at different concentrations. (*n* = 3 ± StDev), Figure S3: Dot flow cytometric plots of A549 cells at 24, 48 and 72 h, when exposed to the control with no treatment, CPP alone, NP-DNA and NP-DNA-CPP, Figure S4: Dot flow cytometric plots of Beas-2B cells at 24, 48 and 72 h, when exposed to the control with no treatment, CPP alone, NP-DNA and NP-DNA-CPP, Figure S5: Flow cytometric plots of cell cycle analysis of A549 cells. On the top panel, on the left, cells were firstly gated to remove debris using SSC x FSC. Sequentially, on the top right, cells were gated to select single cells using FL2-A x FL2-H. The plots on the bottom panel show the cell cycle histogram of (from left to right) cell, NaCl:SF, NP-DNA and NP-DNA-CPP, Figure S6: Flow cytometric plots of cell cycle analysis of Beas-2B cells. On the top panel, on the left, cells were firstly gated to remove debris using SSC x FSC. Sequentially, on the top right, cells were gated to select single cells using FL2-A x FL2-H. The plots on the bottom panel show the cell cycle histogram of (from left to right) cell, NaCl:SF, NP-DNA and NP-DNA-CPP, Figure S7: (A) Quantification of DNA fragmentation using Fiji ImageJ from the confocal images (*n* = 3; ± StDev; **p* < 0.05; ***p* < 0.01); and Confocal Microscopy images of the nucleus of cells exposed to NP coated with CPP (NP-CPP; large image) or control (SF:NaCl; inner image) in both (B) A549 and (C) Beas-2B cells.
