# Supplementary material for: Nanotoxicologic Effects of PLGA Nanoparticles Formulated with a Cell-Penetrating Peptide: Searching for a Safe pDNA Delivery System for the Lungs
Source: Pharmaceutics. 2019 Jan 3;11(1):12. doi: 10.3390/pharmaceutics11010012 (PMC6359528; doi:10.3390/pharmaceutics11010012)
Supplement: Supplementary file 1 [file pharmaceutics-11-00012-s001.pdf]

pharmaceutics

MDPI

Supplementary Materials: Nanotoxicologic Effects of PLGA Nanoparticles Formulated with a Cell-Penetrating Peptide: Searching for a Safe pDNA Delivery System for the Lungs

Larissa Gomes dos Reis, Wing-Hin Lee, Maree Svolos, Lyn Margaret Moir, Rima Jaber, Norbert Windhab, Paul Michael Young and Daniela Traini

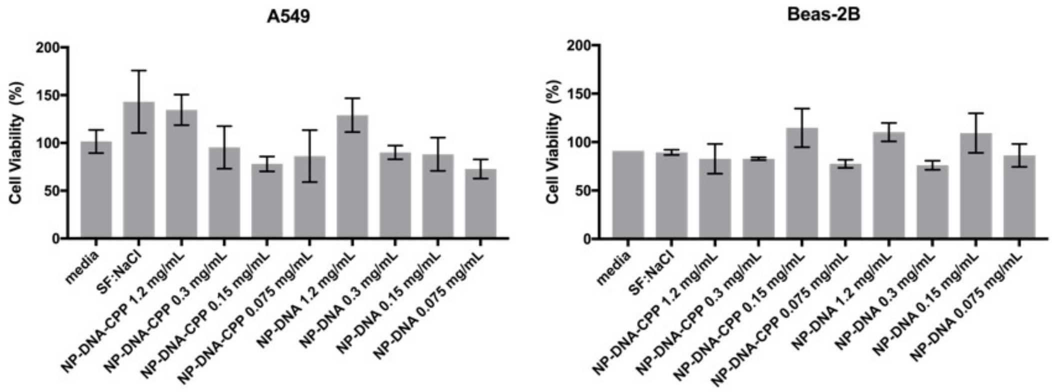

Figure S1: Cytotoxicity of NP-DNA and NP-DNA-CPP in (left panel) A549 and (right panel) Beas-2B cells.

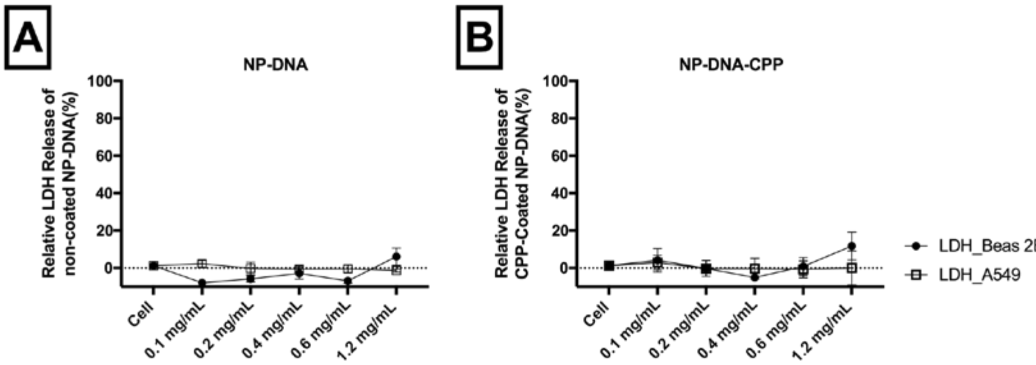

Figure S2: Membrane integrity, via LDH Leakage assay, of both Beas-2B (circles) and A549 cells (squares) exposed to (**A**) NPs or (**B**) NPs-CPP at different concentrations. ($n = 3 \pm$ StDev).

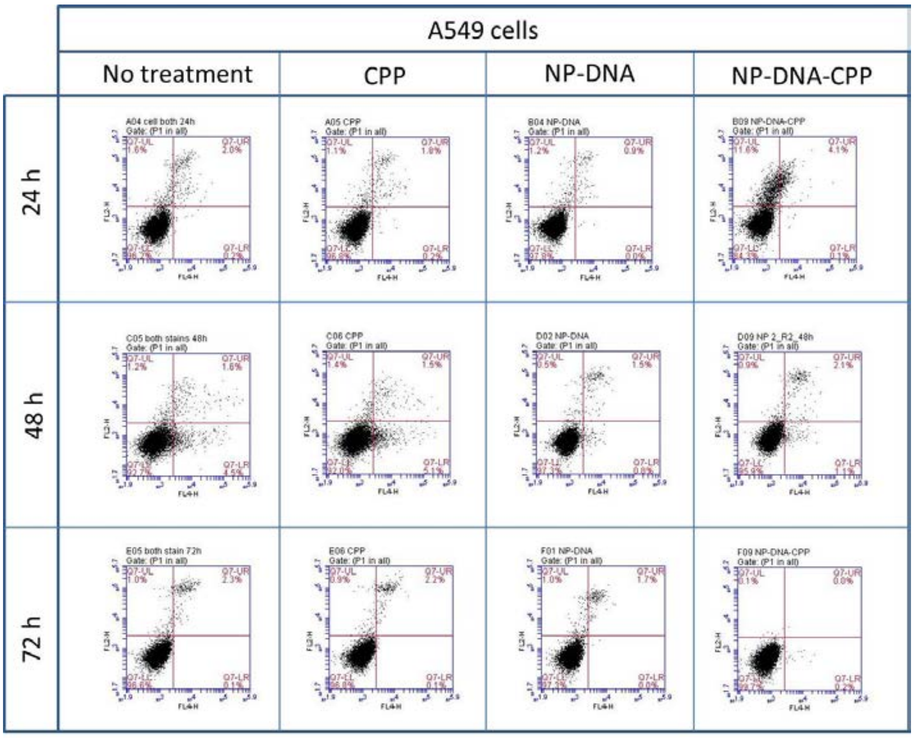

Figure S3: Dot flow cytometric plots of A549 cells at 24, 48 and 72 h, when exposed to the control with no treatment, CPP alone, NP-DNA and NP-DNA-CPP.

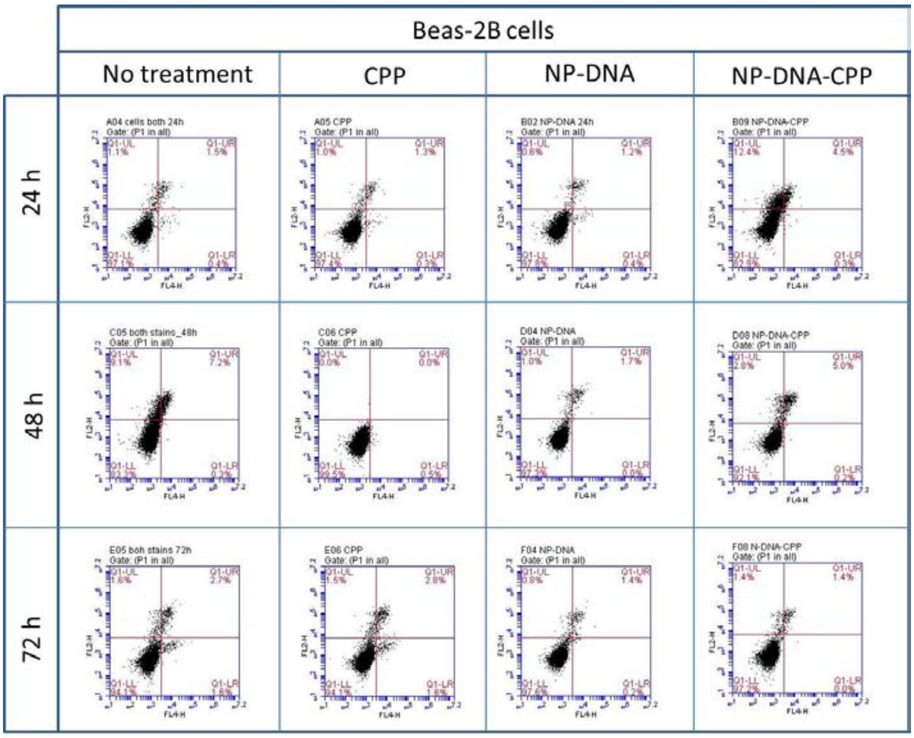

Figure S4: Dot flow cytometric plots of Beas-2B cells at 24, 48 and 72 h, when exposed to the control with no treatment, CPP alone, NP-DNA and NP-DNA-CPP.

A549 plots cell cycle

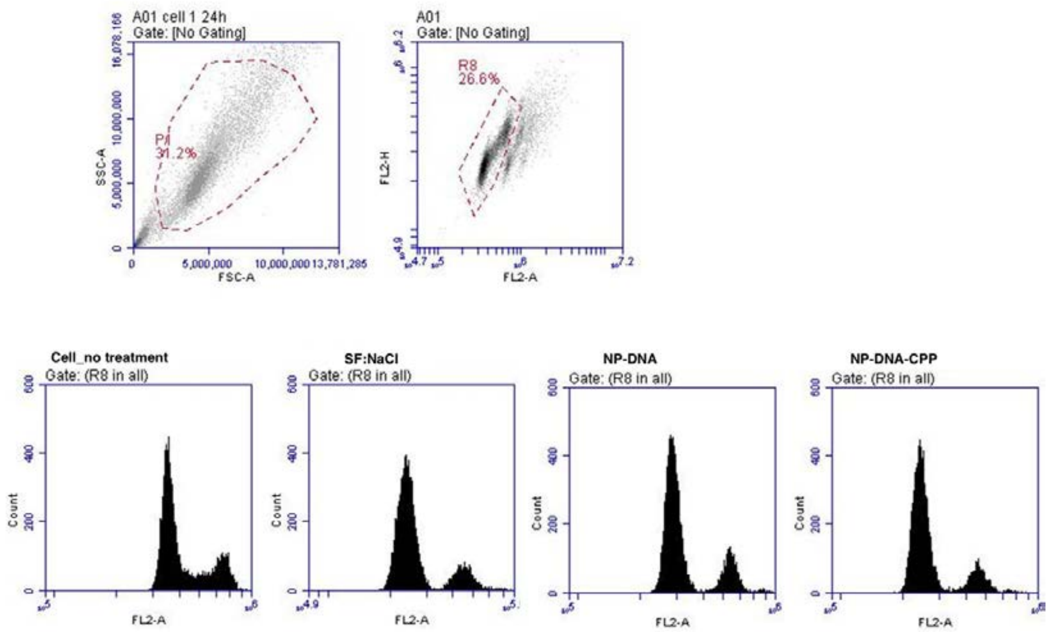

Figure S5: Flow cytometric plots of cell cycle analysis of A549 cells. On the top panel, on the left, cells were firstly gated to remove debris using SSC x FSC. Sequentially, on the top right, cells were gated to select single cells using FL2-A x FL2-H. The plots on the bottom panel show the cell cycle histogram of (from left to right) cell, NaCl:SF, NP-DNA and NP-DNA-CPP.

Beas-2B plots cell cycle

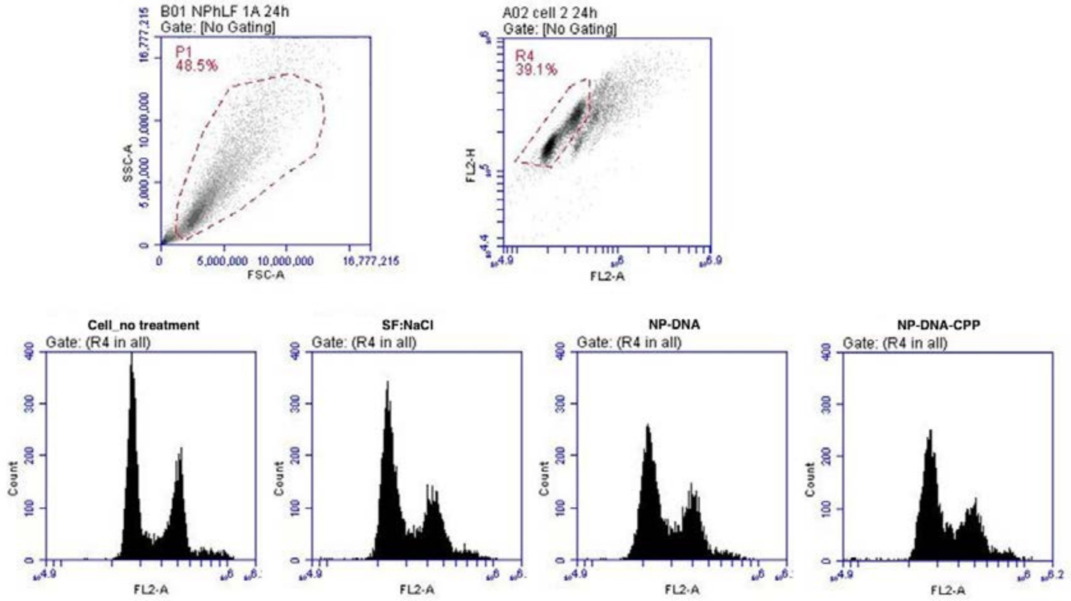

Figure S6: Flow cytometric plots of cell cycle analysis of Beas-2B cells. On the top panel, on the left, cells were firstly gated to remove debris using SSC x FSC. Sequentially, on the top right, cells were gated to select single cells using FL2-A x FL2-H. The plots on the bottom panel show the cell cycle histogram of (from left to right) cell, NaCl:SF, NP-DNA and NP-DNA-CPP.

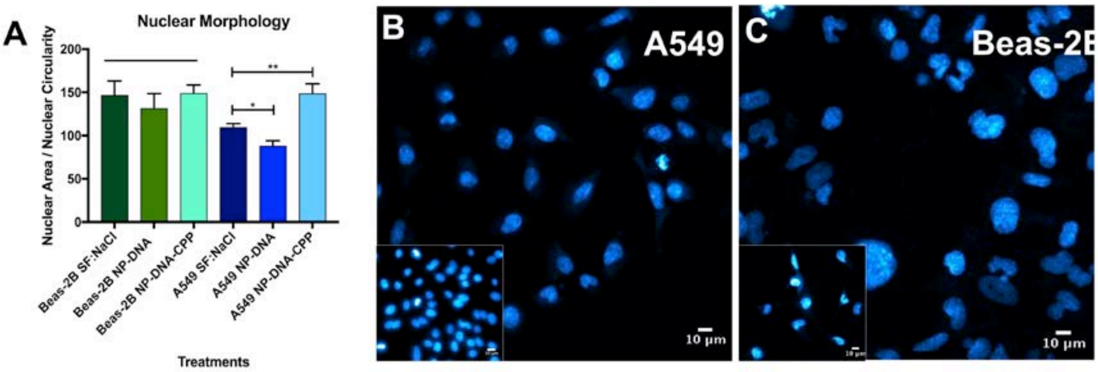

Figure S7: (**A**) Quantification of DNA fragmentation using Fiji ImageJ from the confocal images (n = 3; ± StDev; *P<0.05; **P<0.01); and Confocal Microscopy images of the nucleus of cells exposed to NP coated with CPP (NP-CPP; large image) or control (SF:NaCl; inner image) in both (**B**) A549 and (**C**) Beas-2B cells.
